# Blocking NMDA-Receptors in the Pigeon’s Medial Striatum Impairs Extinction Acquisition and Induces a Motoric Disinhibition in an Appetitive Classical Conditioning Paradigm

**DOI:** 10.3389/fnbeh.2019.00153

**Published:** 2019-07-08

**Authors:** Meng Gao, Roland Pusch, Onur Güntürkün

**Affiliations:** Biopsychology, Institute of Cognitive Neuroscience, Faculty of Psychology, Ruhr University Bochum, Bochum, Germany

**Keywords:** extinction, avian, striatum, NMDA receptors, appetitive, disinhibition

## Abstract

The medial striatum of birds resembles the mammalian dorsal striatum, which plays a key role in the extinction of learned behavior. To uncover the variant and invariant neural properties of extinction learning across species, we use pigeons as an animal model in an appetitive extinction paradigm. Here, we targeted a medial sub-region of the pigeon’s striatum that receives executive, visual and motor pallial projections. By locally antagonizing the *N*-methyl-D-aspartate (NMDA) receptors through 2-Amino-5-phosphonovalerianacid (APV) during extinction, we observed an unspecific disinhibition effect, namely an increase in conditioned pecking to a rewarded control stimulus. In addition, blocking the NMDA receptors substantially deteriorated the extinction acquisition, implying that the pigeons still responded vigorously to the CS- even without food reward during extinction. After correcting for the unspecific effect of APV, the impaired extinction acquisition remained significant, which leads to the assumption that the delayed extinction effect is possibly caused by deficits in the updating of value coding of altered reward contingencies. Also, the APV-induced disinhibition seems to result from local hyperactivity that primarily drives actions towards cues of high appetitive value. The overall correspondence of our results with those from mammals suggests common neural substrates of extinction and highlights the shared functionality of the avian and mammalian dorsal striatum despite 300 million years of independent evolution.

## Introduction

Classically, the basal ganglia had always been associated with motor function. Meanwhile, it is increasingly understood that this system of substructures constitutes the core for a variety of learning, memory, and action selection processes. Since they receive cortical and subcortical projections carrying executive, limbic, sensory, and motivational information, these nuclei are well positioned to foster behavioral strategies to guide motor output in order to achieve favorable outcomes. Thereby, the striatum as a key component of the basal ganglia serves as the main entry gate to the basal ganglia connectivity loop. Numerous studies have indicated that striatal activation coincides with several neuropsychiatric disorders such as post-traumatic stress disorder or substance abuse (Graybiel and Rauch, 2000; Goh and Peterson, 2012; Goodman et al., 2012, 2014; Everitt and Robbins, 2013; Gillan and Robbins, 2014). A thorough understanding of the dorsal striatum memory functions may help to unravel the underlying neural mechanisms for these human psychopathologies, and to further advance their treatment strategies. Therefore, it is of paramount importance to examine not only how the dorsal striatum modulates memory formation, but also how it might be potentially involved in the extinction of learned behavior.

Extinction of learned responses is as important for adaptive behavior as initial acquisition. During extinction, a conditioned stimulus (CS) appears repeatedly without the unconditioned stimulus, or the reinforcer. Consequently, it causes the reduction of the previously learned conditioned response. Evidences suggest that extinction involves partial erasure of the original learning (Rescorla, 2004), as well as the formation of a new memory trace (Bouton et al., 2006). The existence of this new memory trace can be demonstrated by two phenomena: spontaneous recovery (the recovery of the extinguished response caused by a passage of time) and renewal (the recovery of the extinguished response induced by changing the context from the extinction phase to the test phase). Until now, the importance of the mammalian dorsal striatum in the extinction of learned habit behavior has been repeatedly revealed by lesion experiments with monkeys (e.g., Butters and Rosvold, 1968) and rats (e.g., Thullier et al., 1996; Goodman et al., 2016). Studies discovered that it is mostly the dorsolateral (DLS) (e.g., Goodman et al., 2017) and not the dorsomedial part of the striatum (DMS) (Dunnett and Iversen, 1981) that modulates habit memory extinction. Furthermore, post-extinction DLS inactivation impairs memory consolidation after extinction training (Campus et al., 2015). More specifically, it has been shown that NMDA receptors of DLS are the key components which participate in extinguishing habit responses during extinction (Ghasemzadeh et al., 2009; Goodman et al., 2017). Apart from a role in habit memory, the dorsal striatum in mammals also participates in Pavlovian fear conditioning (Ferreira et al., 2003, 2008). However, its involvement in fear extinction seems to be contradictory. A metabolic mapping study showed an elevated glucose consumption in the dorsal striatum during fear extinction in rats (Barrett et al., 2003), whereas a lesion study revealed no effect in Pavlovian fear extinction after excitotoxic lesions in both DLS and DMS (Wendler et al., 2014).

In order to uncover invariant properties of extinction learning in evolutionary distantly related animals, we are using pigeons as an animal model in an appetitive Pavlovian conditioning task. Pigeons are an excellent model system for learning and memory. Behaviorally, they can work with large numbers of visual stimuli while keeping track of individual reward contingencies, and adapting their responses accordingly (Güntürkün et al., 2014). Although the forebrain of birds and mammals differs in many respects, both vertebrate classes have homologous structures like the striatum and the hippocampus, as well as non-homologous, but functionally equivalent structures like the nidopallium caudolaterale (NCL) which is comparable to the mammalian prefrontal cortex (PFC) (Güntürkün, 2012; Güntürkün and Bugnyar, 2016). Several studies started to uncover the neural basis of extinction learning in birds. It is indicated that the prefrontal-like NCL, the hippocampus and the (pre)motor arcopallium, are crucial in the consolidation of extinction memory in birds (Lengersdorf et al., 2014; Gao et al., 2018). Also, acquisition of extinction memory engages the NCL and the amygdala via NMDA receptors in these regions (Lissek and Güntürkün, 2005; Lengersdorf et al., 2015; Gao et al., 2018). In addition, transiently inactivating the nidopallium frontolaterale (NFL), one of the pigeons’ associative visual areas impairs extinction acquisition and perturbs context processing during extinction (Gao et al., 2019). Altogether, the avian neural substrates of extinction learning exhibit comparable characteristics to those in mammals, although their last common ancestor lived ca. 300 million years ago.

In the present study, we attempt to answer the question whether the avian striatum participates in extinguishing learned behavior, since the mammalian striatum plays a crucial role in extinction learning (for a review see Goodman and Packard, 2018). Avian and mammalian striata are considered to be homologous to each other. Both are enriched in dopamine receptors (Sun and Reiner, 2000) and are innervated by dopamine fibers from the substantia nigra pars compacta and the ventral tegmental area (Bottjer, 1993; Casto and Ball, 1994; Wynne and Güntürkün, 1995). Both have neuropils that are rich in acetylcholine and cholinesterase (Reiner et al., 1994), and both have abundant GABAergic medium-sized neurons with spiny dendrites, which contain either substance P or enkephalin (Grisham and Arnold, 1994; Medina and Reiner, 1995; Reiner et al., 1998). In addition, the developing embryonic dorsal striatum in both birds and mammals expresses the same Dlx1/2 genes (Rubenstein et al., 1994; Puelles et al., 2000), which indicates the same developmental neuroepithelial origin.

Anatomical studies indicate that the avian dorsal striatum is composed of medial (StM) and lateral striatum (StL) (Veenman et al., 1995; Reiner et al., 1998, 2004). Despite the many similarities between avian and mammalian striatum, there is possibly no homology in their respective striatal subregions (Reiner, 2002). Although the striatum as a whole in birds and mammals projects to both substantia nigra and pallidum, neurons projecting to substantia nigra and pallidum are intermingled throughout the DMS and DLS in mammals (Reiner and Anderson, 1990; Reiner et al., 1998, 2004). By contrast, the StM of birds contains mainly striatonigral projecting neurons, whereas the StL projects primarily to the pallidum (Bottjer, 1993; Reiner et al., 1998, 2004). Therefore, StM and StL together form the avian dorsal striatum, but do not appear to be one-to-one homologous to the DMS and DLS, respectively. In addition, the corticostriatal projections in mammals are topographically organized (Alexander et al., 1986; Alexander and Crutcher, 1990). For example, the DLS is primarily innervated with sensorimotor and motor cortices, while DMS receives efferents from visual, auditory areas as well as associative and prefrontal regions (McGeorge and Faull, 1989). In birds, evidence has shown that pallial input to the avian striatum is as extensive as that in mammals and arises from all major parts of the pallium (Veenman et al., 1995; Reiner, 2002). However, the pallial-striatal projections are intermingled in StM and StL, having no topographical organization (Veenman et al., 1995; Reiner et al., 1998; Reiner, 2002). For example, the StM receives inputs from the sensory, motor, and associative regions (Veenman et al., 1995; Medina and Reiner, 1997; Kröner and Güntürkün, 1999), possibly showing a mixture of DMS and DLS characteristics.

In the present study, we targeted a subregion within the avian StM that receives pallial efferents from the “prefrontal-like” NCL, the visual associativ NFL, and the (pre)motor arcopallium (Veenman et al., 1995; Kröner and Güntürkün, 1999; Letzner et al., 2016). We aim to investigate the role of StM in the course of Pavlovian extinction, since the sources of its pallio-striatal projections are all significantly involved in extinction (Lengersdorf et al., 2014, 2015; Gao et al., 2018). Considering the importance of the rodent dorsal striatal NMDA receptors in extinction learning (Goodman et al., 2017), and the presence of NMDA receptors in the avian striatum (Wada et al., 2004; Herold et al., 2014), we bilaterally injected the NMDA receptor antagonist APV in the target region before extinction. We adopted a well established behavioral paradigm (Rescorla, 2008; Lengersdorf et al., 2014, 2015; Gao et al., 2018) for pigeons to test the hypothesis that the blockade of NMDA receptors in the StM impairs extinction learning.

## Materials and Methods

### Animals

In total, 22 adult homing pigeons (*Columba livia*) from local breeders participated in the study. The animals were individually housed in separate wire-mesh home cages (40 × 40 × 45 cm) in a colony room, where the temperature, humidity and the 12-h-light-dark circle were strictly controlled (lights on at 8 am). Since we adopted a pavlovian conditioning procedure with food reward, all the animals were food deprived prior to training, and maintained at 80–90% of their free-feeding body weight. Water was provided *ad libitum* in their home cages with additional free food on weekends. Subjects were treated in accordance with the German guidelines for the care and use of animals in science and all experimental procedures were approved by a national ethics committee of the State of North Rhine-Westphalia, Germany and were in agreement with the European Communities Council Directive 86/609/EEC concerning the care and use of animals for experimental purposes.

### Surgery

Prior to behavioral training, the animals received chronical implantation of one 26-gauge (10 mm) stainless steel guide cannulas (Plastics One Inc., Roanoke, United States) in each brain hemisphere. For anesthesia, a 7:3 mixture of ketamine (100 g/ml; Pfizer GmbH, Berlin, Germany) and xylazine (20 mg/ml Rompun, Bayer Vital GmbH, Leverkusen, Germany) was injected i.m. with a dosage of 0.075 ml per 100 g body weight. Additional isoflorane (Forane 100%, Abbott GmbH & Co. KG, Wiesbaden, Germany) was applied through a breathing mask to maintain a stable anesthetized state. Body temperature was maintained using a heat pad during surgery.

When reflexes were tested negatively for pain perception, the animals were fixed in a stereotaxic device. With one incision in the skin, the skull was exposed. Craniotomies were performed on both sides with the following coordinates based on the pigeon brain atlas (Karten and Hodos, 1967): AP+10.5 mm, ML ± 2 mm. Under visual inspection, one cannula was inserted vertically into the medial striatum (StM) in each hemisphere (DV+5.3 mm). The abovementioned coordinates were carefully chosen based on previous tracing studies (Veenman et al., 1995; Medina and Reiner, 1997; Kröner and Güntürkün, 1999). We calculated the coordinates with the aim to target a sub-region within the StM that receives efferents from the visual associative NFL, the (pre)motor arcopallium and the NCL since all three regions are involved in extinction learning based on our previous studies (Lengersdorf et al., 2015; Gao et al., 2018, 2019). 3–5 stainless steel micro-screws (Small Parts, Logansports, United States) were drilled into the skull around each cannula as anchors. In the end, dental cement was applied on the cannulas and the screws to secure the cannulas to the implanted positions. After surgery, all animals received analgesic injections with 0.5 ml Carprofen (Rimadyl, 10 mg/ml Pfizer GmbH, Münster, Germany) twice daily on three consecutive days. The recovery period was 7 days in total, where the animals received free food and water. Two days before restarting the behavioral training, they were food deprived again and maintained at 80–90% of their free-feeding body weight.

### Behavioral Apparatus

The same behavioral apparatus was used as in the previous studies (Lengersdorf et al., 2014, 2015; Gao et al., 2018). Briefly, experimental chambers were four skinner boxes of similar shapes (36 × 34 × 36 cm), which were housed in sound-attenuating cubicles (80 × 80 × 80 cm). Opposite to the opening of the skinner boxes, animals could see the stimulus presented on the monitor screen (either Belinea Model No.: 10 15 36 or Philips Model: Brilliance17S1/00) through the transparent pecking key (5 × 5 cm; 12 cm above the floor). Every effective key peck produced a feedback sound. Food was provided by a food hopper, which was positioned at the bottom center directly underneath the pecking key.

The skinner boxes were grouped in two distinct contexts by using different wallpapers (either with 2.5 cm wide vertical brown stripes spaced 5 cm apart on red background or marbling pattern on turquoise background) on the rear and side walls to differentiate between the contexts. Different noise, either white or brown noise (60 dB SPL), was used additionally during training for better distinction of the two contexts. Well distinguishable visual stimuli were used in the study (see section “Behavioral Procedure”). The hardware was controlled by a custom written MATLAB code Matlab (The Mathworks, Natick, MA, United States) using the Biopsychology toolbox (Rose et al., 2008).

### Behavioral Procedure

**FIGURE 1:**
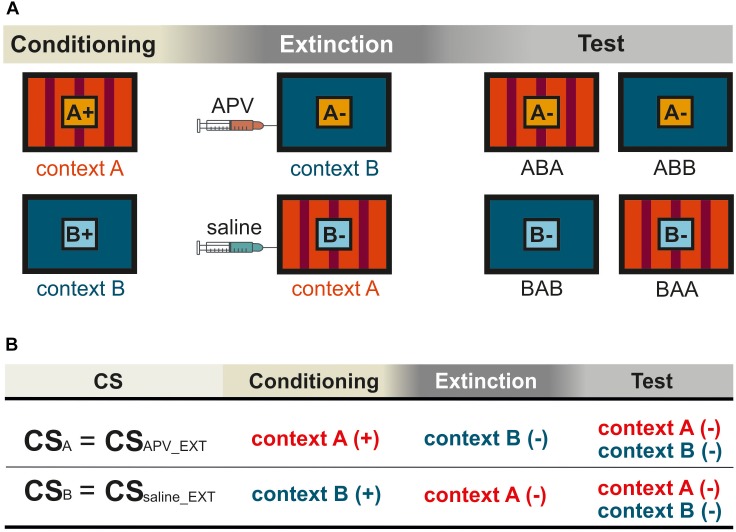
Schematic representation for the within-subject design in the experiment. Pictures show the frontal view of monitor screen in two training chambers A and B. **(A)** The orange and light blue squares with letters A and B show CS_A_ and CS_B_, respectively. The “+” indicates that the CS was rewarded, and the “–” indicates that the CS was not rewarded. Not shown are the “target” (rewarded) and the “non-target” (not rewarded). **(B)** The training histories of the two CSs were illustrated according to **(A)**, with (+) indicating food reward and (–) no food reward. The two CSs were processed under different pharmacological conditions during extinction. For simplification, CS_APV _EXT_ and CS_saline_EXT_ were used to refer to CS_A_ and CS_B_, respectively. The subscript notes of “APV_EXT” and “saline_EXT” indicate the timing of injections that is prior to extinction training. In the experiment, contexts, stimuli, and injection sequences were balanced across subjects. The figures show only one possible example of (1) the sequence in which animals were exposed to contexts A and B, and (2) the sequence in which they received saline and TTX infusions, all of which were counterbalanced across animals.

**FIGURE 2:**
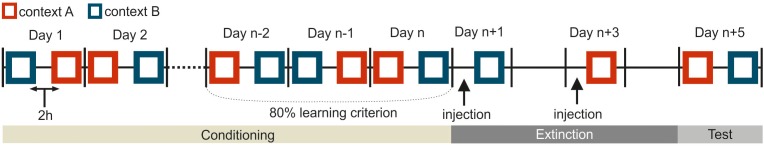
Schematic representation for the experimental procedure in the experiment. This depiction shows only one possible example, and the pretraining I and II are not included. Squares indicate a single training session in one corresponding context (depicted in dark blue or red). The black vertical bars separate consecutive workdays from each other. In the conditioning phase, two sessions were separated 2 h apart from each other on every workday. The conditioning phase was at least 6 days. The specific duration (*n*) depended on how long the pigeons needed to achieve the learning criterion. During the extinction phase on day *n* + 1 and *n* + 3, the animals were trained with one extinction session per day. The black arrows on day *n* + 1 and *n* + 3 indicate the injections of different substances either drug or saline 15 min before extinction training. There was no training the day after the injection to ensure the complete wash out of injected agents from the body system. The subjects were tested in each context on day *n* + 5.

The training procedure was identical to that used in Lengersdorf et al. (2014, 2015), and Gao et al. (2018). Briefly, training was composed of five separate phases: pretraining I, pretraining II, conditioning, extinction and test (Table 1 and Figure 1, 2). Except of the extinction training (described later), animals were trained with two sessions per training day with one session in each context (Figure 2).

**Table 1:** Overview of the experimental procedure.

Phase	Context	# Target (T)	# Non-target (NT)	# CS_A_ or CS_B_
Pretraining I	A	48 × T (+)	–	–
	B	48 × T (+)	–	–
Pretraining II	A	24 × T (+)	12 × NT (-)	–
	B	24 × T (+)	12 × NT (-)	–
Conditioning	A	12 × T (+)	12 × NT (-)	12 × CS_A_ (+)
	B	12 × T (+)	12 × NT (-)	12 × CS_B_ (+)
Extinction	A	24 × T (+)	12 × NT (-)	24 × CS_B_ (-)
	B	24 × T (+)	12 × NT (-)	24 × CS_A_ (-)
Test	A	12 × T (+)	12 × NT (-)	12 × CS_A_ (-) and 12 × CS_B_ (-)
	B	12 × T (+)	12 × NT (-)	12 × CS_A_ (-) and 12 × CS_B_ (-)


*(+) rewarded stimulus; (-) non-rewarded stimulus; CS_*A*_, conditioned stimulus A; CS_*B*_, conditioned stimulus B; –, no stimulus presentation.*

#### Pretraining I and II

In pretraining I, there were 48 trials in each session. In each trial, a stimulus (“target”) was presented for 5 s and followed by 3 s food reward with grain provided by the food hopper. This “target” stimulus was always rewarded, no matter whether the pigeons responded or not. The inter-trial-interval (ITI) was fixed at 48 s. Immediately when the animals achieved the learning criterion with consistent pecking responses in 80% of the trials in both contexts on three consecutive days, the animals entered the second pretraining phase. In pretraining II, in addition to the “target” stimulus, another control stimulus (“non-target”) was introduced. The “non-target” was never rewarded regardless of the response of the animals. Each session consisted of 24 trials of “target” and 12 trials of “non-target” presentations (5 s) in both contexts. The ITI was reduced to 35 s. A minimum of 80% correct responses (pecking response to the “target” and no response to the “non-target”) in both contexts for three consecutive training days were required to enter the conditioning phase.

#### Conditioning

During subsequent conditioning phases, animals were trained with an additional CS in each context. They received training for CS_A_ in context A and CS_B_ in context B (Figure 1A). Each of the three stimuli (“target,” “non-target” and the corresponding CS) was presented for 5 s in 12 trials with 36 trials in total per session. The CS and “target” presentations were rewarded by 3 s of food access via the food hopper, while the “non-target” was not rewarded. Specifically, the duration of the conditioning phase was dependent on how long the pigeons needed to achieve the learning criterion with 80% correct responses for all stimuli across three consecutive days.

#### Extinction

The extinction phase consisted of 4 days in total. The two extinction sessions were scheduled 48 h apart from each other (Figure 1A, 2). The pigeons received an extinction session in each context where the corresponding CS was no longer paired with food reward. 15 min before extinction training, the pigeons were micro-infused bilaterally with either 1 μl of 5 μg/μl APV (Lissek and Güntürkün, 2003; Lengersdorf et al., 2015; Gao et al., 2018) dissolved in 0.9% saline (Tocris Cookson Ltd., Bristol, United Kingdom) or 1 μl 0.9 % saline (B. Braun Melsungen AG., Germany). The order of injections (APV or saline) was randomized across subjects and contexts. There was one day without experimental intervention between each extinction session to ensure a complete wash out of the injected substances from the body. Extinction sessions took place in the two contexts with one session in each context (Figure 1A): the CSs were presented without reward in the other context in which they hadn’t been presented in the conditioning phase (Figure 1A). Simply, the CS_A_ that was previously presented and rewarded in context A, was now presented without reward in context B during extinction. Similarly, the CS_B_ that had been used previously in context B during conditioning, was now presented in context A during extinction without reward. In each extinction session, animals received the corresponding non-rewarded CS- (24 trials), rewarded “target” (24 trials) and non-rewarded “non-target” (12 trials). Again, only the target stimulus was rewarded for 3 s. The order of contexts was randomized across subjects.

#### Test

In the final testing phase and 48 h after the second extinction session, responses to all four stimuli were tested under drug free conditions (Figure 2 and Table 1). Each stimulus was presented for 5 s and for 12 times in each context with 2 h between the two testing sessions. One session contained 48 trials in total. In the testing phase only the target stimulus was rewarded.

Overall, our within-subject design (Figure 1) allows each pigeon to be compared with itself for two different conditions under different pharmacological manipulations. For example, CS_A_ was acquired in context A, extinguished in context B, and tested in both A and B. Thus, we had two conditions, ABA and ABB. Renewal can be observed in the ABA condition, while spontaneous recovery is visible in ABB. For CS_B_ the BAB was the same as ABA, and the BAA equaled ABB. During extinction, the two CSs were processed under different pharmacological conditions. Therefore, CS-_APV _EXT_ refers to the CS under the effect of drug, in this case CS_A_. For simplification, CS_APV _EXT_ is also used to refer to the CS responses in conditioning and testing phases (CS+_APV _EXT_ in conditioning and CS-_APV _EXT_ in testing), although conditioning and testing sessions were conducted drug-free. The same applies for CS_saline_EXT_, accordingly. Again, the subscript note of “APV_EXT” and “saline_EXT” indicate the timing of injections that is prior to extinction training. In order to assess the effect of APV on spontaneous recovery, we compared CS-_APV _EXT_ with CS-_saline_EXT_ in condition ABB/BAA within one pigeon. In addition, by comparing CS-_APV _EXT_ with CS-_saline_EXT_ in ABA/BAB, it revealed how the drug affected the renewal. As described above, apart from the two CSs that underwent extinction, we trained the pigeons with two additional control stimuli, the “target” and the “non-target.” The “target” stimulus remained rewarded throughout the whole experiment, whereas the non-target was never rewarded at all (Table 1). The purpose of including the control stimuli was to identify possible non-specific effects induced by APV infusions.

### Histology

After the animals went through all the behavioral experiments, histology was conducted to verify whether the cannulas were positioned in the StM. To prevent blood clots, animals were injected i.m. with 0.1 ml heparine (Rotexmedica GmbH, Trittau, Germany) dissolved in 0.1 ml of 0.9% NaCl before the perfusion procedure. 15 min later, anesthetization was introduced by i.m. injection of equithesin (0.55 ml/100 g body weight). After the animal was tested negatively for pain stimulation, the animal’s circulatory system was transcardially flushed with ca. 500 ml of 0.9% saline (40°C). Subsequently animals were perfused with 1 L 4% paraformaldehyde (VWR Prolabo Chemicals, Leuven, Belgium). After dissection of the brains, they were post-fixed for at least 2 h in paraformaldehyde and 30% sucrose at 4°C. Afterwards it was transferred in 30% sucrose diluted in 0.12 M PBS for 24 h for cryoprotection. Finally, the brains were embedded in 15% Gelatine (Merck KGaA, Darmstadt, Germany) dissolved in 30% sucrose for 12 h fixation in 4% paraformaldehyde, and then preserved in the solution of 30% sucrose and 0.12 M PBS. For the last steps of histology, the brains were cut frontally into 40 μm slices on a microtome (Leica Microsystems GmbH, Nussloch, Germany), and then stained with cresyl violet to reveal the brain structures. The atlas of the pigeon brain from Karten and Hodos (1967) was used to identify the positions of cannulas.

### Data Analysis

The pecking response to “target,” “non-target,” CS_APV_EXT_, and CS_Saline_EXT_ were registered and stored using a custom-built interface and a custom written matlab code. The number of registered responses on the pecking key during a 5 s stimulus presentation were the main dependent variable.

IBM SPSS Statistic (Version 21, IBM Corp., Armonk, NY, United States) and Matlab were used for statistical analysis. The data from the last three training sessions in the conditioning phase were included for statistical analysis. During the extinction session, pecking responses were restructured into six blocks for the target (24 trials) and CS (24 trials) with four consecutive trials constituting one block. While for non-target (12 trials) two consecutive trials constitute one block, and therefore, also form six blocks. In the test phase, we summarized the data from ABA and BAB conditions and named it as ABA for simplification purpose. ABB was used to refer to the conditions of ABB and BAA. Normal distribution was evaluated by Kolmogorov–Smirnov test. Then data sets were analyzed with Repeated Measure ANOVA (RMANOVA). Mauchly’s test was conducted to validate the data sphericity. On occasion of violation of the sphericity, the Greenhouse–Geisser or Huynh–Feld corrections was applied. Importantly, *post hoc* tests were conducted in case of significant factor effects.

## Results

### Histology

**FIGURE 3:**
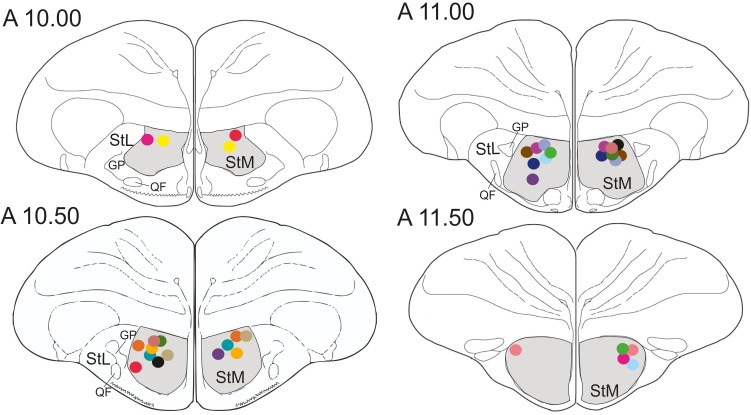
Schematic frontal sections of the pigeon brain showing drug injection sites. Dots represent cannula tips. Each color represents a cannula pair of one pigeon. There were 17 pigeons in the experiment. StM, medial striatum; StL, lateral striatum; GP, globus pallidus; QF, quintofrontal tract. Coordinates are derived from the brain atlas by Karten and Hodos (1967).

In total, 22 pigeons participated in the study. Five pigeons were excluded from further analysis since three animals failed to learn the task after surgery and in two pigeons the cannulas were incorrectly placed. Thus, data from the remaining 17 pigeons were analyzed. In all 17 pigeons cannula implantations were successfully positioned in the StM of both hemispheres, as revealed by histological analysis (Figure 3).

### Learning Speed of the Animals

**FIGURE 4:**
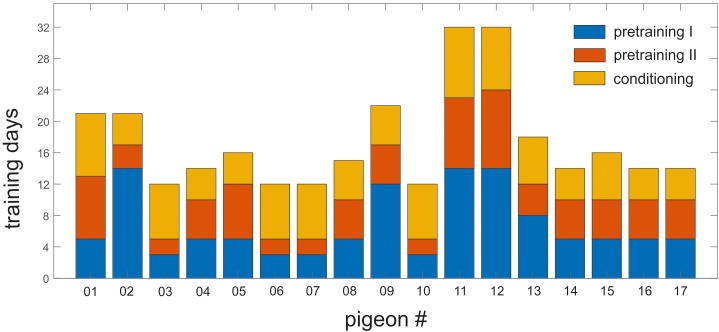
Training history of individual pigeons in pretraining I and II and the conditioning phase. Each bar represents one individual pigeon. *Y*-axis shows the number of training days required to reach the 80% training criterion in all phases. Blue, orange, and yellow represent the pretraining I, pretraining II, and the conditioning phase, respectively. The animals needed minimum 12 days and maximum 32 days to learn the task.

On average, the animals needed 17.29 (±1.44) days of training to achieve the learning criterion of 80% of correct responding for target, non-target and the corresponding CS of each context. This duration includes training phases of pretraining I and II as well as conditioning (Figure 4).

### Conditioning

**FIGURE 5:**
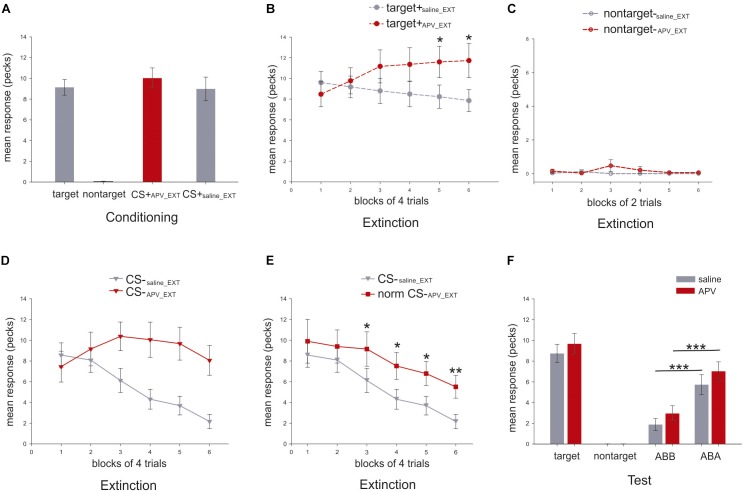
Mean response rates for different stimuli in the present experiment (*N* = 17). *Y*-axis indicates the mean number of pecks during the 5 s stimulus presentation. **(A)** Mean response rates (±SEM) for the three stimuli were calculated in the last three conditioning sessions. **(B)** Mean response rates (±SEM) of the target are shown in dashed line with filled circle for the six blocks under APV (red) and saline (gray) in extinction. **(C)** Mean pecking rates (±SEM) of the non-target, depicted in dashed line with empty circle, are presented for the six blocks under APV (red) and saline (gray) in extinction. **(D)** Mean pecking rates (±SEM) of the CS, shown in solid line with filled triangle, are presented for the six blocks under APV (red) and saline (gray) in extinction. **(E)** Normalized response rates (±SEM) of CS-_APV _EXT_, indicated by solid line with filled square, are depicted against CS-_saline_EXT_ for the six blocks in extinction. **(F)** Mean response rates (±SEM) for the stimuli in the test were presented. Gray and red indicate saline and APV, respectively. Data from both ABA and BAB conditions was summarized together and was labeled as ABA for simplification, and the same was done for ABB and BAA which was named as ABB.

In the last three sessions of conditioning, the mean response to the target (9.1 ± 0.8; mean ± SEM), the CS+_APV_EXT_ (10.1 ± 1.0) and the CS+_saline_EXT_ (9.0 ± 1.1; Figure 5A) did not differ from each other (paired sample *t*-test: target vs. CS+_APV_EXT_: *t*_(16)_ = 1.3, *p* = 0.224, Cohen’s *d* = 0.24; target vs. CS+_saline_EXT_: *t*_(16)_ = 0.3, *p* = 0.796, Cohen’s *d* = 0.03; CS+_APV_EXT_ vs. CS+_saline_EXT_: *t*_(16)_ = 0.92, *p* = 0.373, Cohen’s *d* = 0.24; Figure 5A).

### Extinction

Two-way RMANOVA for both target and CS responding was conducted with two factors, the block and the injection (APV or saline).

For the mean response to the target, neither an effect of injection (two-way RMANOVA, *F*_(1,16)_ = 2.8, *p* = 0.115, $\eta_{p}^{2}$ = 0.15, Figure 5B) nor a block effect (Greenhouse–Geisser correction: *F*_(3.0,47.3)_ = 0.7, *p* = 0.551, $\eta_{p}^{2}$ = 0.04) was observed. However, there was a significant effect for injection × block interaction (*F*_(2.2,35.4)_ = 5.6, *p* = 0.006; $\eta_{p}^{2}$ = 0.26) indicating that animals responded to the target differently under the effects of the two injections during extinction. Specifically, the target pecking responses remained constant under saline injections across six blocks (one-way RMANOVA, *F*_(5,80)_ = 1.7, *p* = 0.147, $\eta_{p}^{2}$ = 0.10) but increased significantly under APV (Greenhouse-Geisser correction: *F*_(2.0,32.2)_ = 3.6, *p* = 0.005, $\eta_{p}^{2}$ = 0.18; Figure 5B). *Post hoc* tests revealed that there were significant differences in fifth (*p* = 0.019, $\eta_{p}^{2}$ = 0.30) and sixth (*p* = 0.024, $\eta_{p}^{2}$ = 0.28) blocks for pecking under APV compared with saline. For pecking to the non-target, there was no effect of injection (two-way RMANOVA, *F*_(1,16)_ = 1.0, *p* = 3.329, $\eta_{p}^{2}$ = 0.06), nor block (Greenhouse–Geisser correction: *F*_(1,16)_ = 1.0, *p* = 0.333, $\eta_{p}^{2}$ = 0.06), nor interaction (Greenhouse–Geisser correction: *F*_(1,16)_ = 1.0, *p* = 0.333, $\eta_{p}^{2}$ = 0.06). Under both injections, the responding to non-target stayed at zero (Figure 5C). The results imply that pigeons’ responses to the target stimulus were significantly affected by injections of APV but not for the non-target stimulus. Therefore, the blockade of NMDA receptors in StM induced a disinhibition of conditioned responding to the control stimulus associated with food reward during extinction.

For pecking response to CS, there was a strong effect of injection (two-way RMANOVA, *F*_(1,16)_ = 12.0, *p* = 0.003, $\eta_{p}^{2}$ = 0.43; Figure 5D), block (*F*_(5,80)_ = 7.4, *p* < 0.001, $\eta_{p}^{2}$ = 0.32) and interaction (Greenhouse–Geisser correction: *F*_(4.5,39.8)_ = 6.27, *p* < 0.001, $\eta_{p}^{2}$ = 0.28; Figure 5D). As just described for the target stimulus, APV seems to induce a disinhibition of pecking to reward-associated cues. To account for this effect and disambiguate it from further effects on learning, we normalized the CS response rates in the APV condition by multiplying an index $\frac{{\mathrm{Tar}}_{\mathrm{Sal}}}{{\mathrm{Tar}}_{\mathrm{APV}}}$ (see Eq. 1), which represents the ratio of target response rates under saline to that under APV.

(1) $$\mathrm{normalized}\;{\mathrm{CS}}_{\mathrm{APV}}=\frac{{\mathrm{Tar}}_{\mathrm{Sal}}}{{\mathrm{Tar}}_{\mathrm{APV}}}\times {\mathrm{CS}}_{\mathrm{APV}}$$

This parameter corrects the CS pecking performance and indicates how the pecking response should manifest without the unspecific response effect to appetitive cues induced by APV. This enables us to detect the effect of APV on extinction learning dynamics. The RMANOVA analysis indicated a strong effect of injection (*F*_(1,16)_ = 7.2, *p* = 0.017, $\eta_{p}^{2}$ = 0.31), and of block (Greenhouse–Geisser correction: *F*_(2.7,42.5)_ = 12.3, *p* < 0.001, $\eta_{p}^{2}$ = 0.44; Figure 5E). For the block effect, we now observe that the normalized pecking responding to CS dropped significantly under both conditions (CS-_saline_EXT_: *F*_(5,80)_ = 11.9, *p* < 0.001, $\eta_{p}^{2}$ = 0.43; normalized CS-_APV_EXT_: *F*_(2.7,43.6)_ = 3.6, *p* = 0.024, $\eta_{p}^{2}$ = 0.18; Figure 5E). However, the decrease under APV was slower than under saline. Specifically, from the third block to the end of the extinction training, normalized responding to CS-_APV_EXT_ was significantly higher than to CS-_saline_EXT_ (third block: *p* = 0.010, $\eta_{p}^{2}$ = 0.35; fourth block: *p* = 0.037, $\eta_{p}^{2}$ = 0.25; fifth block: *p* = 0.031, $\eta_{p}^{2}$ = 0.26; sixth block: *p* = 0.001, $\eta_{p}^{2}$ = 0.48; Figure 5E). After normalization, there was no interaction (*F*_(5,80)_ = 0.7, *p* = 0.606, $\eta_{p}^{2}$ = 0.04; Figure 5E) observed. Importantly, the blockade of the NMDA receptors not only caused a disinhibition of a conditioned response towards a rewarded control stimulus but also slowed down the process of extinction learning to the CS.

### Test

In the test phase, there was no difference between response rates to target+_saline_EXT_ and target+_APV_EXT_ (paired sample *t*-test: *t*_(16)_ = 1.7, *p* = 0.1; Figure 5F).

Because of the training histories in different contexts, the animals should show stronger responses in the conditioning context (ABA) as compared to the extinction context (ABB). This is the hallmark of renewal. Accordingly, we observed a significant renewal under both conditions (paired sample *t*-test, CS-_saline_EXT_ in ABB vs. ABA: *t*_(16)_ = -4.5, *p* < 0.001, Cohen’s *d* = 1.18; CS-_APV_EXT_ in ABB vs. ABA: *t*_(16)_ = -4.4, *p* < 0.001, Cohen’s *d* = 1.18; Figure 5F). Specifically, in the extinction context (ABB), the animals responded equally to the CS-_APV_EXT_ and the CS-_saline_EXT_ (*t*_(16)_ = 1.5, *p* = 0.163, Cohen’s *d* = 0.39; Figure 5F). Similar findings were obtained also in the ABA condition that the pecking to the both CSs did not differ from each other (*t*_(16)_ = 1.2, *p* = 0.236, Cohen’s *d* = 0.33; Figure 5F). Our results indicate that APV injections in the StM prior to the extinction training did not interfere with memory retrieval during testing in the extinction context (ABB) as well as in the conditioning context (ABA).

To examine whether the consolidation process of extinction memory was affected by the injection of APV prior to extinction, we compared the CS responses in the last block of extinction with that in the first four trials of the retrieval test in the extinction context (ABB). Results indicated no significant changes of pecking response to CS-_APV_EXT_ (paired sample *t*-test between normalized CS-_APV_EXT_ of the last block of extinction and the CS-_APV_EXT_ in the beginning of test: *t*_(16)_ = -0.4, *p* = 0.682, Cohen’s *d* = 0.09) and to CS-_saline_EXT_ (*t*_(16)_ = 2.0, *p* = 0.059, Cohen’s *d* = 0.53), implying that the pigeons responded equally from the end of extinction to the beginning of testing in the extinction context. Therefore, the consolidation of the extinction memory was not affected by the APV injection.

## Discussion

The aim of the study was to examine the role of NMDA receptors for extinction learning in the medial striatum of pigeons. For this purpose, local NMDA receptors were blocked with APV during extinction. Consequently, we observed severe deficits in the acquisition of extinction memory, as well as a disinhibition of conditioned responding towards a rewarded control stimulus. These effects will now be discussed.

The target region of the present study receives massive input from the prefrontal-like NCL as well as smaller projections from the visual associative NFL and the (pre)motor arcopallium (Veenman et al., 1995; Kröner and Güntürkün, 1999; Shanahan et al., 2013). The results obtained from the present study showed highly similar patterns to APV-injections in the NCL using the identical experimental procedure (Lengersdorf et al., 2015). This implies that the avian pallial (NCL) → striatal (StM) pathway plays a similar role as the corticostriatal system in mammals. This possibly explains that both studies evince a delayed extinction acquisition and a disinhibition of conditioned pecking to a reward stimulus after blocking NMDA receptors.

### Blocking StM NMDA Receptors Impaired Extinction Acquisition

The observation of a delayed extinction in the present work is in good accordance to studies in other bird species. Excitotoxic lesions in the chick StM delayed extinction of operant pecking without affecting the learned inhibition of pecking the non-rewarded stimulus as well as the operant pecking at a rewarded control stimulus (Ichikawa et al., 2004). Yanagihara et al. (2001) demonstrated that a population of the StM neurons code the chick’s evaluation of instantaneous rewards. Consequently, Ichikawa et al. (2004) propose that delayed extinction after StM lesions might be caused by a disturbed update of value coding of altered reward contingencies during extinction. This might also be one of the key explanations for the impairment in extinction acquisition observed in our study. To date, several studies could reveal a central role of the NCL in encoding reward amount and subjective reward value (Kalenscher et al., 2005; Koenen et al., 2013; Dykes et al., 2018). It is therefore possible that NCL efferents have a strong impact on reward value coding of StM neurons (Yanagihara et al., 2001), which also explains the similarity of the results from our study with that obtained from NCL.

Since the StM receives inputs from the visual, (pre)motor, and prefrontal regions (Veenman et al., 1995; Medina and Reiner, 1997; Kröner and Güntürkün, 1999), it possibly has a mixture of DMS and DLS characteristics. Accordingly, our findings are comparable to the extinction data obtained from mammals (Butters and Rosvold, 1968; Baranov, 1977; Denisova, 1981). Especially, the linkage of the DLS to the extinction of habit learning (Dunnett and Iversen, 1981; Goodman et al., 2017) via local NMDA receptors (Goodman et al., 2017) are well reported. Similarly, there is evidence that neurons in the monkeys’ DMS display stimulus-related activity, which is believed to code for the anticipation of sensory cues that signal reward (Kimura et al., 1993). This finding echoes well with the electrophysiological findings in chick StM neurons (Yanagihara et al., 2001). Furthermore, the present study indicates a specific role of NMDA modulated synaptic plasticity of the avian striatum in extinction. There is evidence showing that activity-dependent long-term synaptic plasticity in the zebra finch striatum is involved in song learning and requires the activation of NMDA receptors (Ding and Perkel, 2004). Similarly, mammalian NMDA receptors in the dorsal striatum are also needed for long-term potentiation, synaptic depotentiation (Calabresi et al., 1992; Li et al., 2009). And the enhancement or impairment of habit memory extinction can be achieved through modulation of NMDA receptors in the DLS (Goodman et al., 2017). Given the highly conserved structure of the amniote basal ganglia across species (for a review see Medina and Reiner, 1995), it is likely that neural mechanisms in the dorsal striatum might operate in birds and mammals in a similar way. Accordingly, our study provides the first evidence that NMDA-dependent synaptic changes in the avian dorsal striatum is involved in Pavlovian extinction learning.

In addition, studies with rodents usually use extended training to ensure habit learning during task acquisition (e.g., Packard and McGaugh, 1996), while our pigeons also received more than thousand trials in total. It is therefore reasonable to believe that the extensive training in our experiment may have induced habit learning as well. Since the avian StM in pigeons has anatomical connections with somatic and (pre)motor areas (Medina and Reiner, 1995; Reiner et al., 1998), antagonizing the NMDA receptors during extinction may deteriorate the extinction dynamics from the habitual aspect of responding in our task. It is therefore reasonable to speculate that the impairment of extinction acquisition observed in the present study may be explained by (1) a disturbed update in value coding propagated via the prefrontal-like NCL, and (2) an impaired extinction of habit responding due to its connections with (pre)motor arcopallium.

### Blocking StM NMDA Receptors Induced a Disinhibition of the Conditioned Responding to a Rewarded Control Stimulus

In the present experiment on the StM and the previous one investigating the role of the NCL (Lengersdorf et al., 2015), a disinhibition of conditioned responding to a rewarded control stimulus was found under the effect of local APV injections during extinction. It has been repeatedly shown that increased responding can be induced by pharmacological administration of NMDA receptor antagonist (Carlsson and Carlsson, 1989; Adams and Moghaddam, 1998; Moghaddam and Adams, 1998; Gainetdinov et al., 2001). At the neurophysiological level, the systemic injection of the non-competitive NMDA receptor antagonist MK801 produces an increased number of disorganized spike bouts of PFC neurons in freely moving rats (Jackson et al., 2004). These disorganized single spikes co-occurred with elevated locomotion and behavioral stereotypy (Jackson et al., 2004). In addition, MK801 injections preferentially reduced the activity of prefrontal GABAergic interneurons, thereby disinhibiting pyramidal neurons and resulting in an elevated excitation of cortical outputs (Homayoun and Moghaddam, 2007). At the synaptic level, administration of various kinds of NMDA receptor antagonists increases the extracellular levels of glutamate (Moghaddam et al., 1997; Adams and Moghaddam, 1998; Moghaddam and Adams, 1998; Lorrain et al., 2003) and dopamine (Carlsson and Carlsson, 1989; Carrozza et al., 1992; Keefe et al., 1992; Martínez-Fong et al., 1992; Lorrain et al., 2003) in PFC and striatum. Importantly, evidence with pigeons also suggests that systemically elevated dopamine receptor activity increases the pecking responses to the stimulus that is predictive of food delivery (Anselme et al., 2018). Jointly, the above-mentioned evidence makes it likely that increased pecking to the rewarded “target” as observed in the current study results from APV-induced hyperactivity. However, this effect was confined to the rewarded “target”, but not to the “non-target.” One possible reason may lie in the stronger appetitive value of the “target” in comparison to the “non-target.” Upon non-rewarded stimulus presentations, the “hyperactive” striatal neurons induced by local APV injection might still be lower than threshold, and therefore, did not induce any changes in behavioral output. That said, it is presently unclear that the hyperactivity results from alterations of motivational or purely motoric processes (Adams and Moghaddam, 1998; Jackson et al., 2004; Gökhan et al., 2017).

## Conclusion

Our results support the hypothesis that NMDA receptors in the avian StM are involved in distinct aspects of extinction learning. By locally injecting APV prior to extinction, the response decrement during extinction acquisition was severely impaired. In addition, we also observed a disinhibited responding to the rewarded control stimulus but not to the non-rewarded one. The resemblance of our data to that from NCL with the same behavioral paradigm hints to the presence of a functional pallial-striatal pathway in the avian brain similar to the prefrontal-striatal network in mammals. Furthermore, the comparative approach denotes the shared functionality of the avian and mammalian dorsal striata in extinction learning and suggests some invariant properties in evolutionary distant vertebrates that derived from the last common ancestor 300 million years ago.

## Data Availability

The datasets generated for this study are available on request to the corresponding author.

## Ethics Statement

This study was carried out in accordance with the German guidelines for the care and use of animals in science and all experimental procedures were approved by the National Ethics Committee of the State of North Rhine-Westphalia, Germany and were in agreement with the European Communities Council Directive 86/609/EEC concerning the care and use of animals for experimental purposes.

## Author Contributions

MG performed the experiments and data analysis. OG conceived the original idea. OG and RP supervised the study. MG wrote the manuscript with the support from OG and RP.

## Conflict of Interest Statement

The authors declare that the research was conducted in the absence of any commercial or financial relationships that could be construed as a potential conflict of interest.

## References

[B1] Adams B. Moghaddam B. (1998). Corticolimbic dopamine neurotransmission is temporally dissociated from the cognitive and locomotor effects of Phencyclidine. *J. Neurosci.* 18 5545–5554. 10.1523/JNEUROSCI.18-14-05545.1998 9651235 PMC6793475

[B2] Alexander G. E. Crutcher M. D. (1990). Functional architecture of basal ganglia circuits: neural substrates of parallel processing. *Trends Neurosci.* 13 266–271. 10.1016/0166-2236(90)90107-L 1695401

[B3] Alexander G. E. DeLong M. R. Strick P. L. (1986). Parallel organization of functionally segregated circuits linking basal ganglia and cortex. *Annu. Rev. Neurosci.* 9 357–381. 10.1146/annurev.ne.09.030186.002041 3085570

[B4] Anselme P. Edeş N. Tabrik S. Güntürkün O. (2018). Long-term behavioral sensitization to apomorphine is independent of conditioning and increases conditioned pecking, but not preference, in pigeons. *Behav. Brain Res.* 336 122–134. 10.1016/J.BBR.2017.08.037 28859998

[B5] Baranov V. V. (1977). Extinction of inhibition following damage to the orbital cortex and ventral portion of the head of the caudate nucleus in dogs. *Zh. Vyssh. Nerv. Deiat. Im. I P Pavlova* 27 196–199. 855482

[B6] Barrett D. Shumake J. Jones D. Gonzalez-Lima F. (2003). Metabolic mapping of mouse brain activity after extinction of a conditioned emotional response. *J. Neurosci. η* 23 5740–5749. 10.1523/jneurosci.23-13-05740.2003 12843278 PMC6741242

[B7] Bottjer S. W. (1993). The distribution of tyrosine hydroxylase immunoreactivity in the brains of male and female zebra finches. *J. Neurobiol.* 24 51–69. 10.1002/neu.480240105 8093477

[B8] Bouton M. E. Westbrook R. F. Corcoran K. A. Maren S. (2006). Contextual and temporal modulation of extinction: behavioral and biological mechanisms. *Biol. Psychiatry* 60 352–360. 10.1016/j.biopsych.2005.12.015 16616731

[B9] Butters N. Rosvold H. E. (1968). Effect of caudate and septal nuclei lesions on resistance to extinction and delayed-alternation. *J. Comp. Physiol. Psychol.* 65(3 PART 1), 397–403. 10.1016/S0021-9940(07)65728-0 4970002

[B10] Calabresi P. Pisani A. Mercuri N. B. Bernardi G. (1992). Long-term potentiation in the striatum is unmasked by removing the voltage-dependent magnesium block of NMDA receptor channels. *Eur. J. Neurosci.* 4 929–935. 10.1111/j.1460-9568.1992.tb00119.x 12106428

[B11] Campus P. Colelli V. Orsini C. Sarra D. Cabib S. (2015). Evidence for the involvement of extinction-associated inhibitory learning in the forced swimming test. *Behav. Brain Res.* 278 348–355. 10.1016/J.BBR.2014.10.009 25448432

[B12] Carlsson M. Carlsson A. (1989). The NMDA antagonist MK-801 causes marked locomotor stimulation in monoamine-depleted mice. *J. Neural Transm.* 75 221–226. 10.1007/BF01258633 2538557

[B13] Carrozza D. P. Ferraro T. N. Golden G. T. Reyes P. F. Hare T. A. (1992). In vivo modulation of excitatory amino acid receptors: microdialysis studies on N-methyl-d-aspartate-evoked striatal dopamine release and effects of antagonists. *Brain Res.* 574 42–48. 10.1016/0006-8993(92)90797-D 1353403

[B14] Casto J. M. Ball G. F. (1994). Characterization and localization of D1 dopamine receptors in the sexually dimorphic vocal control nucleus, area X, and the basal ganglia of european starlings. *J. Neurobiol.* 25 767–780. 10.1002/neu.480250703 8089655

[B15] Denisova A. S. (1981). Effect of electrical stimulation of the somatosensory cortex and caudate nucleus on extinctive inhibition of a food conditioned reflex to sound. *Neurosci. Behav. Physiol.* 11 576–581. 10.1007/BF01186836 7347805

[B16] Ding L. Perkel D. J. (2004). Long-term potentiation in an avian basal ganglia nucleus essential for vocal learning. *J. Neurosci.* 24 488–494. 10.1523/jneurosci.4358-03.2004 14724247 PMC6729982

[B17] Dunnett S. B. Iversen S. D. (1981). Learning impairments following selective kainic acid-induced lesions within the neostriatum of rats. *Behav. Brain Res.* 2 189–209. 10.1016/0166-4328(81)90055-3 7248057

[B18] Dykes M. Klarer A. Porter B. Rose J. Colombo M. (2018). Neurons in the pigeon Nidopallium caudolaterale display value-related activity. *Sci. Rep.* 8:5377. 10.1038/s41598-018-23694-8 29599499 PMC5876356

[B19] Everitt B. J. Robbins T. W. (2013). From the ventral to the dorsal striatum: devolving views of their roles in drug addiction. *Neurosci. Biobehav. Rev.* 37 1946–1954. 10.1016/J.NEUBIOREV.2013.02.010 23438892

[B20] Ferreira T. L. Moreira K. M. Ikeda D. C. Bueno O. F. Oliveira M. G. M. (2003). Effects of dorsal striatum lesions in tone fear conditioning and contextual fear conditioning. *Brain Res.* 987 17–24. 10.1016/S0006-8993(03)03217-7 14499941

[B21] Ferreira T. L. Shammah-Lagnado S. J. Bueno O. F. A. Moreira K. M. Fornari R. V. Oliveira M. G. M. (2008). The indirect amygdala-dorsal striatum pathway mediates conditioned freezing: insights on emotional memory networks. *Neuroscience* 153 84–94. 10.1016/j.neuroscience.2008.02.013 18367339

[B22] Gainetdinov R. R. Mohn A. R. Bohn L. M. Caron M. G. (2001). Glutamatergic modulation of hyperactivity in mice lacking the dopamine transporter. *Proc. Natl. Acad. Sci. U.S.A.* 98 11047–11054. 10.1073/pnas.191353298 11572967 PMC58681

[B23] Gao M. Lengersdorf D. Stüttgen M. C. Güntürkün O. (2018). NMDA receptors in the avian amygdala and the premotor arcopallium mediate distinct aspects of appetitive extinction learning. *Behav. Brain Res.* 343 71–82. 10.1016/j.bbr.2018.01.026 29378293

[B24] Gao M. Lengersdorf D. Stüttgen M. C. Güntürkün O. (2019). Transient inactivation of the visual-associative nidopallium frontolaterale (NFL) impairs extinction learning and context encoding in pigeons. *Neurobiol. Learn. Mem.* 158 50–59. 10.1016/j.nlm.2019.01.012 30664941

[B25] Ghasemzadeh M. B. Mueller C. Vasudevan P. (2009). Behavioral sensitization to cocaine is associated with increased glutamate receptor trafficking to the postsynaptic density after extended withdrawal period. *Neuroscience* 159 414–426. 10.1016/J.NEUROSCIENCE.2008.10.027 19105975

[B26] Gillan C. M. Robbins T. W. (2014). Goal-directed learning and obsessive-compulsive disorder. *Philos. Trans. R. Soc. Lond. B Biol. Sci.* 369:20130475. 10.1098/rstb.2013.0475 25267818 PMC4186229

[B27] Goh S. Peterson B. S. (2012). Imaging evidence for disturbances in multiple learning and memory systems in persons with autism spectrum disorders. *Dev. Med. Child Neurol.* 54 208–213. 10.1111/j.1469-8749.2011.04153.x 22269006

[B28] Goodman J. Leong K. C. Packard M. G. (2012). Emotional modulation of multiple memory systems: implications for the neurobiology of post-traumatic stress disorder. *Rev. Neurosci.* 23 627–643. 10.1515/revneuro-2012-0049 23001314

[B29] Goodman J. Marsh R. Peterson B. S. Packard M. G. (2014). Annual research review: the neurobehavioral development of multiple memory systems - Implications for childhood and adolescent psychiatric disorders. *J. Child Psychol. Psychiatry* 55 582–610. 10.1111/jcpp.12169 24286520 PMC4244838

[B30] Goodman J. Packard M. G. (2018). The role of the dorsal striatum in extinction: a memory systems perspective. *Neurobiol. Learn. Mem.* 150 48–55. 10.1016/j.nlm.2018.02.028 29501803

[B31] Goodman J. Ressler R. L. Packard M. G. (2016). The dorsolateral striatum selectively mediates extinction of habit memory. *Neurobiol. Learn. Mem.* 136 54–62. 10.1016/J.NLM.2016.09.012 27663194

[B32] Goodman J. Ressler R. L. Packard M. G. (2017). Enhancing and impairing extinction of habit memory through modulation of NMDA receptors in the dorsolateral striatum. *Neuroscience* 352 216–225. 10.1016/J.NEUROSCIENCE.2017.03.042 28377177

[B33] Gökhan N. Neuwirth L. S. Meehan E. F. (2017). The effects of low dose MK-801 administration on NMDA_R_ dependent executive functions in pigeons. *Physiol. Behav.* 173 243–251. 10.1016/j.physbeh.2017.02.009 28192131

[B34] Graybiel A. M. Rauch S. L. (2000). Toward a neurobiology of obsessive-compulsive disorder. *Neuron* 28 343–347. 10.1016/S0896-6273(00)00113-6 11144344

[B35] Grisham W. Arnold A. P. (1994). Distribution of GABA-like immunoreactivity in the song system of the zebra finch. *Brain Res.* 651 115–122. 10.1016/0006-8993(94)90686-6 7922557

[B36] Güntürkün O. (2012). The convergent evolution of neural substrates for cognition. *Psychol. Res.* 76 212–219. 10.1007/s00426-011-0377-9 21881941

[B37] Güntürkün O. Bugnyar T. (2016). Cognition without cortex. *Trends Cogn. Sci.* 20 291–303. 10.1016/j.tics.2016.02.001 26944218

[B38] Güntürkün O. Stüttgen M. C. Manns M. (2014). Pigeons as a model species for cognitive neuroscience. *E-Neuroforum* 20 86–92. 10.1515/s13295-014-0057-5 17184744

[B39] Herold C. Bingman V. P. Ströckens F. Letzner S. Sauvage M. Palomero-Gallagher N. (2014). Distribution of neurotransmitter receptors and zinc in the pigeon (*Columba livia*) hippocampal formation: a basis for further comparison with the mammalian hippocampus. *J. Comp. Neurol.* 522 2553–2575. 10.1002/cne.23549 24477871

[B40] Homayoun H. Moghaddam B. (2007). NMDA receptor hypofunction produces opposite effects on prefrontal cortex interneurons and pyramidal neurons. *J. Neurosci. η* 27 11496–11500. 10.1523/JNEUROSCI.2213-07.2007 17959792 PMC2954603

[B41] Ichikawa Y. Izawa E. I. Matsushima T. (2004). Excitotoxic lesions of the medial striatum delay extinction of a reinforcement color discrimination operant task in domestic chicks; a functional role of reward anticipation. *Cogn. Brain Res.* 22 76–83. 10.1016/j.cogbrainres.2004.08.001 15561503

[B42] Jackson M. E. Homayoun H. Moghaddam B. (2004). NMDA receptor hypofunction produces concomitant firing rate potentiation and burst activity reduction in the prefrontal cortex. *Proc. Natl. Acad. Sci. U.S.A.* 101 8467–8472. 10.1073/pnas.93.10.4693 15159546 PMC420417

[B43] Kalenscher T. Windmann S. Diekamp B. Rose J. Güntürkün O. Colombo M. (2005). Single units in the pigeon brain integrate reward amount and time-to-reward in an impulsive choice task. *Curr. Biol.* 15 594–602. 10.1016/J.CUB.2005.02.052 15823531

[B44] Karten H. J. Hodos W. (1967). *A Stereotaxic Atlas of the Brain of the Baboon (Papio)*. Baltimore, MD: The Johns Hopkins University Press, 10.1212/WNL.19.8.808-a

[B45] Keefe K. A. Zigmond M. J. Abercrombie E. D. (1992). Extracellular dopamine in striatum: influence of nerve impulse activity in medial forebrain bundle and local glutamatergic input. *Neuroscience* 47 325–332. 10.1016/0306-4522(92)90248-Z 1353620

[B46] Kimura M. Aosaki T. Ishida A. (1993). Neurophysiological aspects of the differential roles of the putamen and caudate nucleus in voluntary movement. *Adv. Neurol.* 60 62–70. 8380529

[B47] Koenen C. Millar J. Colombo M. (2013). How bad do you want it η Reward modulation in the avian nidopallium caudolaterale. *Behav. Neurosci.* 127 544–554. 10.1037/a0033551 23895064

[B48] Kröner S. Güntürkün O. (1999). Afferent and efferent connections of the caudolateral neostriatum in the pigeon (*Columba livia*): a retro-and anterograde pathway tracing study. *J. Comp. Neurol.* 407 228–260. 10.1098/rstb.2002.1238 10213093

[B49] Lengersdorf D. Marks D. Uengoer M. Stüttgen M. C. Güntürkün O. (2015). Blocking NMDA-receptors in the pigeon’s “prefrontal” caudal nidopallium impairs appetitive extinction learning in a sign-tracking paradigm. *Front. Behav. Neurosci.* 9:85. 10.3389/fnbeh.2015.00085 25918502 PMC4394694

[B50] Lengersdorf D. Stüttgen M. C. Uengoer M. Güntürkün O. (2014). Transient inactivation of the pigeon hippocampus or the nidopallium caudolaterale during extinction learning impairs extinction retrieval in an appetitive conditioning paradigm. *Behav. Brain Res.* 265 93–100. 10.1016/j.bbr.2014.02.025 24569011

[B51] Letzner S. Simon A. Güntürkün O. (2016). Connectivity and neurochemistry of the commissura anterior of the pigeon (*Columba livia*). *J. Comp. Neurol.* 524 343–361. 10.1002/cne.23858 26179777 PMC5049482

[B52] Li P. Li Y.-H. Han T.-Z. (2009). NR2A-containing NMDA receptors are required for LTP induction in rat dorsolateral striatum in vitro. *Brain Res.* 1274 40–46. 10.1016/j.brainres.2009.04.016 19376094

[B53] Lissek S. Güntürkün O. (2003). Dissociation of extinction and behavioral disinhibition: the role of NMDA receptors in the pigeon associative forebrain during extinction. *J. Neurosci. η* 23 8119–8124. 10.1523/jneurosci.23-22-08119.2003 12954874 PMC6740504

[B54] Lissek S. Güntürkün O. (2005). Out of context: NMDA receptor antagonism in the avian “prefrontal cortex” impairs context processing in a conditional discrimination task. *Behav. Neurosci.* 119 797–805. 10.1037/0735-7044.119.3.797 15998201

[B55] Lorrain D. S. Baccei C. S. Bristow L. J. Anderson J. J. Varney M. A. (2003). Effects of ketamine and N-methyl-D-aspartate on glutamate and dopamine release in the rat prefrontal cortex: modulation by a group II selective metabotropic glutamate receptor agonist LY379268. *Neuroscience* 117 697–706. 10.1016/S0306-4522(02)00652-8 12617973

[B56] Martínez-Fong D. Rosales M. G. Góngora-Alfaro J. L. Hernández S. Aceves G. (1992). NMDA receptor mediates dopamine release in the striatum of unanesthetized rats as measured by brain microdialysis. *Brain Res.* 595 309–315. 10.1016/0006-8993(92)91065-M 1361416

[B57] McGeorge A. J. Faull R. L. M. (1989). The organization of the projection from the cerebral cortex to the striatum in the rat. *Neuroscience* 29 503–537. 10.1016/0306-4522(89)90128-0 2472578

[B58] Medina L. Reiner A. (1995). Neurotransmitter organization and connectivity of the basal ganglia in Vertebrates: implications for the evolution of basal ganglia. *Brain Behav. Evol.* 46 235–258. 10.1159/000113277 8564466

[B59] Medina L. Reiner A. (1997). The efferent projections of the dorsal and ventral pallidal parts of the pigeon basal ganglia, studied with biotinylated dextran amine. *Neuroscience* 81 773–802. 10.1016/S0306-4522(97)00204-2 9316028

[B60] Moghaddam B. Adams B. Verma A. Daly D. (1997). Activation of glutamatergic neurotransmission by Ketamine: a novel step in the pathway from NMDA receptor blockade to dopaminergic and cognitive disruptions associated with the prefrontal cortex. *J. Neurosci.* 17 2921–2927. 10.1523/JNEUROSCI.17-08-02921.1997 9092613 PMC6573099

[B61] Moghaddam B. Adams B. W. (1998). Reversal of Phencyclidine effects by a group II metabotropic glutamate receptor agonist in rats. *Science* 281 1349–1352. 10.1126/SCIENCE.281.5381.1349 9721099

[B62] Packard M. G. McGaugh J. L. (1996). Inactivation of hippocampus or caudate nucleus with lidocaine differentially affects expression of place and response learning. *Neurobiol. Learn. Mem.* 65 65–72. 10.1006/NLME.1996.0007 8673408

[B63] Puelles L. Kuwana E. Puelles E. Bulfone A. Shimamura K. Keleher J. (2000). Pallial and subpallial derivatives in the embryonic chick and mouse telencephalon, traced by the expression of the genes Dlx-2, Emx-1, Nkx-2.1*, Pax-*6, and Tbr-1. *J. Comp. Neurol.* 424 409–438. 10.1002/1096-9861(20000828)424:3<409::AID-CNE3<3.0.CO;2-7 10906711

[B64] Reiner A. (2002). Functional circuitry of the avian basal ganglia: implications for basal ganglia organization in stem amniotes. *Brain Res. Bull.* 57 513–528. 10.1016/S0361-9230(01)00667-0 11923021

[B65] Reiner A. Anderson K. D. (1990). The patterns of neurotransmitter and neuropeptide co-occurrence among striatal projection neurons: conclusions based on recent findings. *Brain Res. Rev.* 15 251–265. 10.1016/0165-0173(90)90003-7 1981156

[B66] Reiner A. Karle E. J. Anderson K. D. Medina L. (1994). “Catecholaminergic perikarya and fibers in the avian nervous system,” in *Phylogeny and Development of Catecholamine Systems in the CNS of Vertebrates*, eds Smeets W. J. A. J. Reiner A. (Cambridge: Cambridge University Press), 135–181.

[B67] Reiner A. Medina L. Veenman C. L. (1998). Structural and functional evolution of the basal ganglia in vertebrates. *Brain Res. Rev.* 28 235–285. 10.1016/S0165-0173(98)00016-2 9858740

[B68] Reiner A. Perkel D. J. Bruce L. L. Butler A. B. Csillag A. Kuenzel W. (2004). Revised nomenclature for avian telencephalon and some related brainstem nuclei. *J. Comp. Neurol.* 473 377–414. 10.1002/cne.20118 15116397 PMC2518311

[B69] Rescorla R. A. (2004). Spontaneous recovery varies inversely with the training-extinction interval. *Learn. Behav.* 32 401–408. 10.3758/BF03196037 15825882

[B70] Rescorla R. A. (2008). Within-subject renewal in sign tracking. *Q. J. Exp. Psychol.* 61 1793–1802. 10.1080/17470210701790099 18609366

[B71] Rose J. Otto T. Dittrich L. (2008). The biopsychology-toolbox: a free, open-source Matlab-toolbox for the control of behavioral experiments. *J. Neurosci. Methods* 175 104–107. 10.1016/j.jneumeth.2008.08.006 18765252

[B72] Rubenstein J. L. Martinez S. Shimamura K. Puelles L. (1994). The embryonic vertebrate forebrain: the prosomeric model. *Science* 266 578–580. 10.1126/SCIENCE.7939711 7939711

[B73] Shanahan M. Bingman V. P. Shimizu T. Wild M. Güntürkün O. (2013). Large-scale network organization in the avian forebrain: a connectivity matrix and theoretical analysis. *Front. Comput. Neurosci.* 7:89. 10.3389/fncom.2013.00089 23847525 PMC3701877

[B74] Sun Z. Reiner A. (2000). Localization of dopamine D1A and D1B receptor mRNAs in the forebrain and midbrain of the domestic chick. *J. Chem. Neuroanat.* 19 211–224. 10.1016/S0891-0618(00)00069-7 11036238

[B75] Thullier F. Lalonde R. Mahler P. Joyal C. C. Lestienne F. (1996). Dorsal striatal lesions in rats. 2: effects on spatial and non-spatial learning. *Arch. Physiol. Biochem.* 104 307–312. 10.1076/apab.104.3.307.12895org/10.1076/apab.104.3.307.12895 8793022

[B76] Veenman C. L. Wild J. M. Reiner A. (1995). Organization of the avian “corticostriatal” projection system: a retrograde and anterograde pathway tracing study in pigeons. *J. Comp. Neurol.* 354 87–126. 10.1002/cne.903540108 7615877

[B77] Wada K. Sakaguchi H. Jarvis E. D. Hagiwara M. (2004). Differential expression of glutamate receptors in avian neural pathways for learned vocalization. *J. Comp. Neurol.* 476 44–64. 10.1002/cne.20201 15236466 PMC2517240

[B78] Wendler E. Gaspar J. C. C. Ferreira T. L. Barbiero J. K. Andreatini R. Vital M. A. B. F. (2014). The roles of the nucleus accumbens core, dorsomedial striatum, and dorsolateral striatum in learning: performance and extinction of Pavlovian fear-conditioned responses and instrumental avoidance responses. *Neurobiol. Learn. Mem.* 109 27–36. 10.1016/j.nlm.2013.11.009 24291572

[B79] Wynne B. Güntürkün O. (1995). Dopaminergic innervation of the telencephalon of the pigeon (*Columba Livia*) - a study with antibodies against tyrosine-hydroxylase and dopamine. *J. Comp. Neurol.* 357 446–464. 10.1002/cne.903570309 7673478

[B80] Yanagihara S. Izawa E. I. Koga K. Matsushima T. (2001). Reward-related neuronal activities in basal ganglia of domestic chicks. *Neuroreport* 12 1431–1435. 10.1097/00001756-200105250-00027 11388424

